# p66Shc in sheep preimplantation embryos: Expression and regulation of oxidative stress through the manganese superoxide dismutase-reactive oxygen species metabolic pathway

**DOI:** 10.5713/ab.22.0402

**Published:** 2023-02-27

**Authors:** Tong Zhang, Jiaxin Zhang, Ruilan Li

**Affiliations:** 1 School of Medicine, Shanxi Datong University, Datong, Shanxi, 037009, China; 2 Key Laboratory of Animal Genetics, Breeding and Reproduction, Inner Mongolia Autonomous Region, Hohhot, Inner Mongolia, 010018, China; 3 Institute of Reproductive Medicine of Shanxi Datong University, Datong, Shanxi, 037009, China; 4 College of Animal Science, Inner Mongolia Agricultural University, Hohhot, Inner Mongolia, 010018, China

**Keywords:** Embryo, Manganese Superoxide Dismutase (MnSOD), Oxidative Stress, Reactive Oxygen Species (ROS), Sheep

## Abstract

**Objective:**

p66Shc, a 66 kDa protein isoform encoded by the proto-oncogene SHC, is an essential intracellular redox homeostasis regulatory enzyme that is involved in the regulation of cellular oxidative stress, apoptosis induction and the occurrence of multiple age-related diseases. This study investigated the expression profile and functional characteristics of p66Shc during preimplantation embryo development in sheep.

**Methods:**

The expression pattern of p66Shc during preimplantation embryo development in sheep at the mRNA and protein levels were studied by quantitative real-time polymerase chain reaction (RT-qPCR) and immunofluorescence staining. The effect of p66Shc knockdown on the developmental potential were evaluated by cleavage rate, morula rate and blastocyst rate. The effect of p66Shc deficiency on reactive oxygen species (ROS) production, DNA oxidative damage and the expression of antioxidant enzymes (e.g., catalase and manganese superoxide dismutase [MnSOD]) were also investigated by immunofluorescence staining.

**Results:**

Our results showed that p66Shc mRNA and protein were expressed in all stages of sheep early embryos and that p66Shc mRNA was significantly downregulated in the 4- to 8-cell stage (p<0.05) and significantly upregulated in the morula and blastocyst stages after embryonic genome activation (EGA) (p<0.05). Immunofluorescence staining showed that the p66Shc protein was mainly located in the peripheral region of the blastomere cytoplasm at different stages of preimplantation embryonic development. Notably, serine (Ser36)-phosphorylated p66Shc localized only in the cytoplasm during the 2- to 8-cell stage prior to EGA, while phosphorylated (Ser36) p66Shc localized not only in the cytoplasm but also predominantly in the nucleus after EGA. RNAi-mediated silencing of p66Shc via microinjection of p66Shc siRNA into sheep zygotes resulted in significant decreases in p66Shc mRNA and protein levels (p<0.05). Knockdown of p66Shc resulted in significant declines in the levels of intracellular ROS (p<0.05) and the DNA damage marker 8-hydroxy-2′-deoxyguanosine (p<0.05), markedly increased MnSOD levels (p<0.05) and resulted in a tendency to develop to the morula stage.

**Conclusion:**

These results indicate that p66Shc is involved in the metabolic regulation of ROS production and DNA oxidative damage during sheep early embryonic development.

## INTRODUCTION

Sheep *in vitro* fertilization–embryo transfer (IVF–ET) technology is an important reproductive technology. Many research institutions and enterprises use sheep IVF–ET to efficiently produce offspring on a large-scale and in batches [1,2]. However, this technique has always faced the limitations of the poor *in vitro* maturation quality of sheep oocytes, weak embryonic development ability, low embryo production efficiency and unstable effects of embryo transfer. Oxidative stress (OS) caused by reactive oxygen species (ROS) is one of the main factors that threatens the acquisition and maintenance of oocyte and embryo developmental potential during *in vivo* processes and *in vitro* manipulation/development [3,4]. From a literature review, it is evident that many animal studies, including studies on sheep and other livestock species, have shown that gametes and embryos cultured in nonphysiological culture systems are susceptible to environmental OS [5,6]. Excessive production of ROS due to activation of OS-regulating genes *in vitro* culture environment results in an imbalance of redox homeostasis, exceeding the antioxidant defence capacity of the embryo and triggering apoptosis, necrosis, or permanent cell cycle arrest [7,8]. Therefore, it is important to understand and improve the ability of embryos to resist OS from the perspective of redox homeostasis-regulated genes.

p66Shc is a 66 kDa protein isoform encoded by the proto- oncogene Src homologous-collagen homologue (SHC). Furthermore, p66Shc, as an important sensor protein for the regulation of intracellular redox homeostasis, plays a crucial role in regulating ROS-mediated OS, apoptosis and age-related diseases. The N-terminal CH2 region of p66Shc contains a serine phosphorylation site, Ser36, and the phosphorylation activation of this critical serine (Ser36) appears to be a key regulatory mechanism for the apoptotic activity and OS response of p66Shc [9,10]. The phenomenon in which early embryonic development tends to stop at a certain stage is called developmental arrest, which depends on the species; for example, mouse embryos arrest at the 2-cell stage, human and pig embryos arrest at the 4- to 8-cell stage, cattle and sheep embryos arrest at the 8- to 16-cell stage, and rabbit embryos arrest at the morula stage [11,12]. Arsenic can significantly induce developmental arrest and upregulation of p66Shc expression in mouse 2-cell embryos. p66Shc RNA interference results in significant decreases in ROS levels, a reduction in arsenic-induced 2-cell arrest and enhancement of embryonic developmental competence [13]. In addition, loss-of-function studies using RNA interference have shown that p66Shc-induced OS is associated with increased ROS levels in bovine embryos exposed to stress-inducing environmental conditions [14]. Our previous studies have also shown that the poor *in vitro* developmental capacity of oocytes and early embryos is associated with abnormal expression of p66Shc, mitochondrial dysfunction, and imbalance of redox homeostasis [15,16]. These findings suggest that p66Shc is involved in mediating early embryonic cleavage arrest and is critical for subsequent preimplantation development.

Currently, studies involving the regulatory mechanisms of redox homeostasis in early embryos are still in the exploratory stage. Based on our existing research, in this study, we intended to explore the temporally and spatially specific expression of the *p66Shc* gene during preimplantation embryo development in sheep at the mRNA and protein levels. Microinjection of p66Shc siRNA into the cytosol reduced the level of p66Shc at the zygote phase. The effect of p66Shc knockdown on the developmental potential of sheep embryos, the production of ROS, the production of the DNA oxidative damage marker 8-hydroxy-2′-deoxyguanosine (8-OHdG) and the expression of antioxidant enzymes (e.g., catalase and manganese superoxide dismutase [MnSOD]) were investigated. Our results show that p66Shc mRNA and protein, as well as p66Shc phosphorylated at Ser36, show specific expression patterns at particular stages of early embryonic development. RNA interference-mediated knockdown of p66Shc not only improved the developmental potential of early embryos but also decreased intracellular ROS levels, reduced the accumulation of DNA damage and increased the content of MnSOD. These results confirm that the expression and distribution of p66Shc at special stages are closely related to the developmental regulation of early preimplantation embryos. Deletion of p66Shc increases embryonic resistance to OS and developmental potential, suggesting that p66Shc participates in the regulation of OS by mediating the ROS metabolism pathway.

## MATERIALS AND METHODS

### Oocyte collection and *in vitro* maturation

Sheep isolated ovaries were collected from a local slaughterhouse, stored in physiological saline containing 1% (v/v) penicillin–streptomycin in a vacuum flask, and transported to the laboratory within 2 to 3 h at 30°C to 35°C. Cumulus–oocyte complexes (COCs) were aspirated from 3 to 6 mm antral follicles using sterile 10 mL syringes with 18-gauge needles. The COCs with more than two layers of intact cumulus cells and homogeneous ooplasm were selected with a stereomicroscope. A total of 3,431 oocytes were used in this study. The COCs were cultured for 24 h in maturation medium consisting of Medium 199 (with Earle’s salts, L-glutamine, and 2.2 g/L sodium bicarbonate; 11150-059; Gibco, Grand Island, NY, USA) supplemented with 10% (v/v) fetal bovine serum (FBS) (Cat No. 10099-141; Gibco, USA), 10 mg/mL Folltropin-V (FSH; Vetoquinol, Lavaltrie, QC, Canada), 10 mg/mL Lutropin-V (LH; Vetoquinol, Canada), 1 mg/mL β-oestradiol (E-8875; Sigma-Aldrich, Shanghai, China), 100 mM cysteamine (M-9768; Sigma-Aldrich, China), and 1% (v/v) penicillin–streptomycin. A group of 50 COCs were placed in a 4-well culture dish (Nunc cat. 176740; Thermo Scientific, Roskilde, Denmark) that contained 600 mL of medium covered with 300 mL of mineral oil and incubated in a humidified environment containing 5% CO_2_ in air.

### *In vitro* fertilization and embryo culture

*In vitro* fertilization and embryo culture to the blastocyst stage were performed as described previously [17]. Briefly, sheep semen was prepared by a ‘swim-up’ procedure. Matured oocytes were partially denuded in 0.1% hyaluronidase by gentle pipetting and transferred into fertilization medium. Then, the oocytes were cocultured with motile spermatozoa for 20 h in a 38.6°C humidified 5% CO_2_ incubator. Denuded and presumptive zygotes were transferred to synthetic oviductal fluid supplemented with 1% (v/v) basal medium Eagle-essential amino acids, 1% (v/v) minimum essential medium-nonessential amino acids, 1 mM glutamine and 3 mg/mL bovine serum albumin and cultured at 38.6°C in a humidified environment with a 90% N_2_, 5% CO_2_, 5% O_2_ atmosphere. Embryos from different developmental stages were collected at the indicated times for use.

### Microinjection

To attenuate endogenous p66Shc mRNA expression, siRNA against p66Shc was microinjected into the cytoplasm of sheep zygote-stage oocytes. According to the reference sequence in GenBank (Accession number: XM_012132297.2). Three p66Shc siRNAs and nonspecific siRNAs were synthesized by GenePharma (Shanghai, China). All siRNA sequences used in the present study are shown in Table 1. Microinjection was performed using an Eppendorf microinjector in TCM199 supplemented with 2% (v/v) FBS and 7.5 μg/mL cytochalasin B (CB) on the heating stage of an inverted microscope (Carl Zeiss, Gottingen, Germany). Approximately 10 pL of siRNA solution (25 μM) was microinjected into the cytoplasm of zygotes, and three control groups (no injection, RNase-free water injection and nonspecific siRNA injection) were designed to test the potential effects of the microinjection technique and siRNA toxicity on embryonic development.

### Real-time quantitative polymerase chain reaction

The procedures used for total RNA extraction, cDNA synthesis and real-time RT–quantitative polymerase chain reaction (qPCR) to quantify the transcript abundance of MII-stage oocytes and early embryo samples (n = 3 pools of 30 oocytes/embryos per group) were performed as described previously [16]. Embryos were obtained at the following hours after IVF: zygote, 16 h; two-cell, 36 h; four-cell, 46 h; eight-cell, 72 h; morula, 108 h; blastocyst, 168 h. β-Actin was quantified in parallel as a reference gene to normalize the gene expression data. The primer sequences are shown in Table 2. Negative control reactions lacking template DNA were run in parallel. Relative gene expression was calculated by the 2^−ΔΔCt^ method.

### Immunofluorescence staining and confocal analysis

Immunofluorescence staining was performed as described previously [16]. Oocytes or embryos were fixed with 4% paraformaldehyde for 15 min. Then, the fixed oocytes or embryos were permeabilized with 1% (v/v) Triton X-100 in phosphate-buffered saline (PBS) for 25 min at 37°C and blocked in 5% normal goat serum for 1 h at room temperature. The oocytes or embryos were incubated in primary antibody diluted in Antibody Dilution Buffer/Wash (PBS + 1% normal goat serum + 0.005% Triton X-100) at 4°C overnight in a humidified chamber. The primary antibodies used were anti-p66Shc (1:100; Proteintech, Wuhan, China), anti-phospho-Ser36-p66Shc (1:100; Abcam, Shanghai, China), anti-8-OHdG (1:100; Santa Cruz Biotechnology, Shanghai, China), anti-MnSOD (1:100; Abcam, China) and anti-catalase (1:100; Proteintech, China). After washing with 0.01% (w/v) polyvinyl alcohol in PBS, the samples were incubated in secondary antibodies diluted in Antibody Dilution Buffer/Wash within a dark humidified chamber at 4°C overnight. The oocytes or embryos were counterstained with 0.5 μg/mL 4′, 6-diamidino-2-phenylindole (DAPI) for 15 minutes and transferred to a 35-mm confocal dish and observed under the Olympus FluoView FV10i confocal laser scanning microscope system (Olympus, Tokyo, Japan).

### Detection of reactive oxygen species

Embryos from defined stages were collected and incubated with 2,7-dichlorodihydrofluorescein diacetate at a final concentration of 10 μM/mL for 30 min before being imaged using a confocal laser scanning microscope. The fluorescence signal was detected, each set of experiments (30 embryos per group) was repeated three times, and the level of ROS was analysed using ImageJ software.

### Statistical analysis

Each experiment was performed with at least three biological replicates. Statistical analyses were performed using SPSS software (version 17.0). Differences between two groups were analysed by independent-samples t tests, and multiple comparison tests were performed via one-way analyses of variance followed by Student–Newman–Keuls test. The data are expressed as the means±standard errors of the means, and p-values of less than 0.05 were considered to indicate statistical significance.

## RESULTS

### Expression profile and spatial pattern of p66Shc and serine-36 phosphorylation of p66Shc in sheep early preimplantation embryos

**Figure 1:**
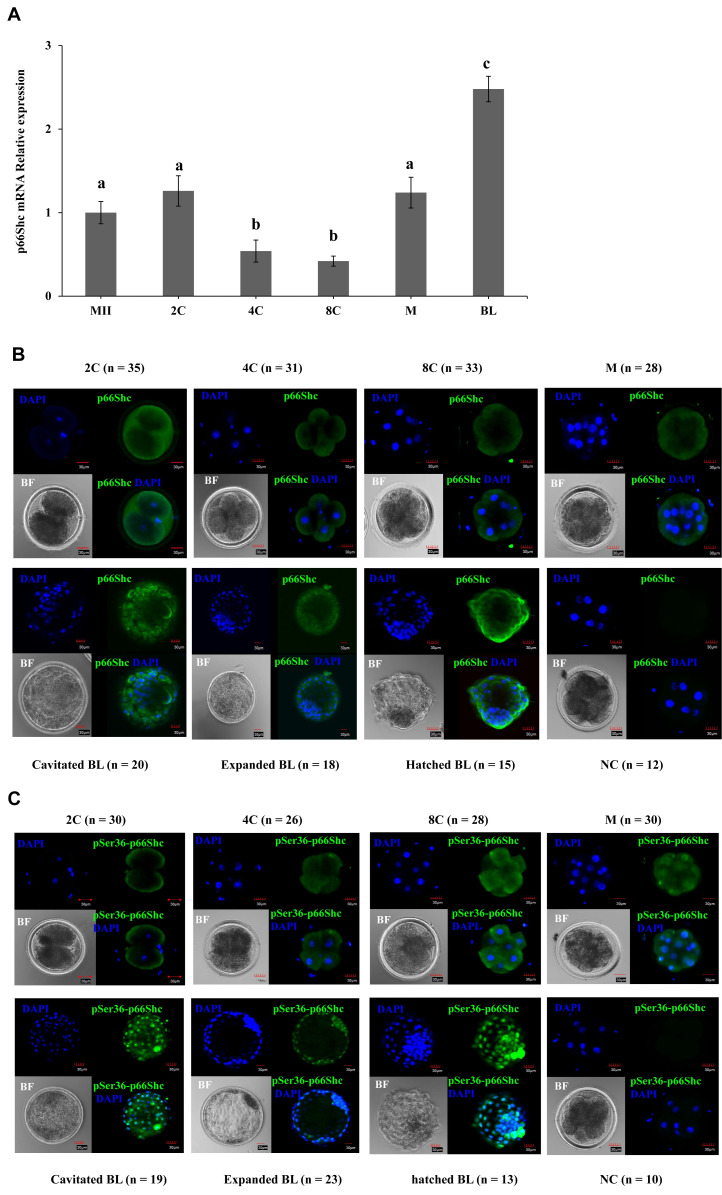
Expression abundance and spatial localization patterns of the p66Shc gene in the early stage of sheep preimplantation embryo development. (A) Quantitative real-time RT-polymerase chain reaction (qRT-PCR) was performed to examine the relative transcript abundance of p66Shc with three replicates of pools of 30 embryos per stage. ^a–c^ p<0.05. (B) The expression abundance and spatial localization pattern of p66Shc protein in embryos at each stage were examined by immunofluorescence staining combined with confocal microscopy. Representative confocal images are shown: 2-cell embryo; 4-cell embryo; 8-cell; morula; cavitated blastocyst; expanded blastocyst; hatched blastocyst, primary antibody omitted. Green = p66Shc, blue = 4′, 6-diamidino-2-phenylindole (DAPI). Scale bar = 30 μm. (C) Immunofluorescence and confocal microscopy for phosphorylated Ser36 p66Shc protein was performed per stage of preimplantation embryo. Representative confocal images are shown: 2-cell embryo; 4-cell embryo; 8-cell; morula; cavitated blastocyst; expanded blastocyst; hatched blastocyst, primary antibody omitted. Green = serine (Ser36) phosphorylated p66Shc, blue = DAPI. Scale bar = 30 μm. Expression abundance and spatial localization patterns of the p66Shc gene in the early stage of sheep preimplantation embryo development. (A) Quantitative real-time RT-polymerase chain reaction (qRT-PCR) was performed to examine the relative transcript abundance of p66Shc with three replicates of pools of 30 embryos per stage. ^a–c^ p<0.05. (B) The expression abundance and spatial localization pattern of p66Shc protein in embryos at each stage were examined by immunofluorescence staining combined with confocal microscopy. Representative confocal images are shown: 2-cell embryo; 4-cell embryo; 8-cell; morula; cavitated blastocyst; expanded blastocyst; hatched blastocyst, primary antibody omitted. Green = p66Shc, blue = 4′, 6-diamidino-2-phenylindole (DAPI). Scale bar = 30 μm. (C) Immunofluorescence and confocal microscopy for phosphorylated Ser36 p66Shc protein was performed per stage of preimplantation embryo. Representative confocal images are shown: 2-cell embryo; 4-cell embryo; 8-cell; morula; cavitated blastocyst; expanded blastocyst; hatched blastocyst, primary antibody omitted. Green = serine (Ser36) phosphorylated p66Shc, blue = DAPI. Scale bar = 30 μm.

To gain better insights into the functional role of p66Shc in sheep early preimplantation embryos, the temporal and spatial dynamics of p66Shc and phosphorylated (Ser36) p66Shc were detected during preimplantation embryo development. The mRNA and protein levels of p66Shc are presented at all stages. RT–qPCR results indicated that p66Shc mRNA exhibits an inverted bell curve expression pattern during preimplantation embryonic development. p66Shc mRNA expression was remarkably downregulated at the 4- to 8-cell stage and significantly upregulated at the morula stage just after embryonic genome activation (EGA), but p66Shc mRNA was steadily expressed from the metaphase II (MII) stage to the 2-cell stage (Figure 1A).

The results of immunocytofluorescence staining revealed that p66Shc protein was predominantly localized in the peripheral region of the cytoplasm of blastomeres at all stages of preimplantation embryo development (Figure 1B). The cellular localization of phosphorylated (Ser36) p66Shc during preimplantation embryo development was determined. Strikingly, the colocalization of phosphorylated (Ser36) p66Shc with a specific antibody and DAPI staining of the nucleus clearly revealed that phosphorylated Ser36 p66Shc was not only located in the cytoplasmic areas but also prevalently localized in the nuclear areas from the cavitated blastocyst to hatched blastocyst stage. However, phosphorylated Ser36 p66Shc only displayed cytoplasmic localization from the 2-cell to the 8-cell stage before EGA (Figure 1C). These results suggest that the specific expression pattern of the *p66Shc* gene during early embryonic development is critical for embryonic development.

### Evaluation of the efficiency of microinjection for siRNA-mediated knockdown of p66shc

**Figure 2:**
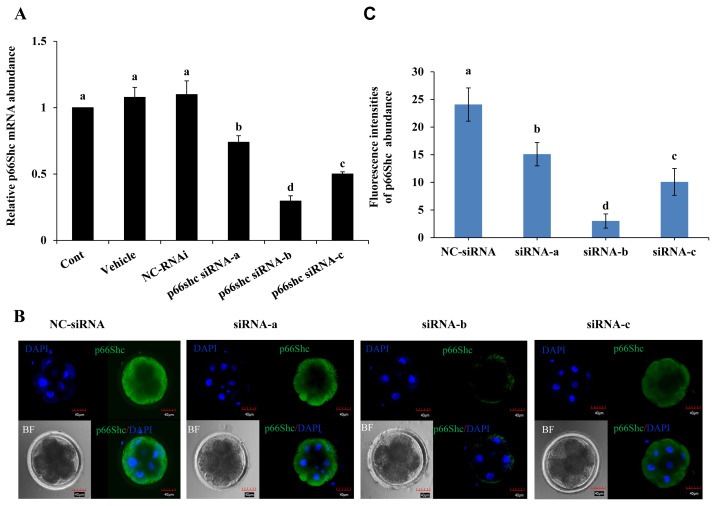
Evaluation of the efficiency of microinjection of siRNA targeting the p66Shc gene at the zygotic stage. (A) Real-time quantitative polymerase chain reaction (qRT-PCR) was used to detect the expression abundance of p66Shc mRNA in four- and eight-cell embryos in groups microinjected with siRNA-a, siRNA-b, or siRNA-c and in the uninjected group (control group), the RNase-free water (vehicle)-injected group and the negative control siRNA (NC-siRNA)-injected group. (B) Representative confocal images of the expression abundance of p66Shc protein in four- and eight-cell embryos after microinjection of siRNA-a, siRNA-b, siRNA-c and the negative control siRNA (NC-siRNA). Green = p66Shc, blue = 4′, 6-diamidino-2-phenylindole (DAPI). Scale bar = 40 μm. All data are shown as the mean±standard error of the mean. ^a–d^ Different lowercase letters indicate significant differences (p<0.05). (C) Quantification intensity of p66Shc in four- and eight-cell embryos following RNAi-mediated knockdown of p66Shc after microinjection of siRNA-a, siRNA-b, siRNA-c, or the negative control siRNA (NC-siRNA) (n = 30 for each group).

To further elucidate the role of p66Shc in preimplantation embryo development, specific siRNA molecules (siRNA-a, siRNA-b, siRNA-c) targeting the *p66Shc* gene were designed and injected using microinjection techniques. The siRNA molecules were injected into the cytoplasm of sheep zygotes by microinjection, while no injection, RNase-free water (vehicle) and negative control siRNA molecules (NC-siRNA) were used as controls. The RT–qPCR results showed that the transcription of p66Shc mRNA at the 4- to 8-cell stage after microinjection of siRNA-a, siRNA-b, or siRNA-c was significantly lower (p<0.05) than that in the control groups, and the interference efficiency was 26%, 71%, or 50%, respectively. siRNA-b was the most efficient molecule for knockdown of p66Shc. However, there was no significant difference among the group microinjected with RNase-free water, the group microinjected with NC-siRNA and the noninjected control group (p>0.05) (Figure 2A).

To further verify the interference efficiency after cytoplas mic microinjection of p66Shc siRNA molecules, the protein levels of p66Shc in 4- to 8-cell-stage embryos were detected by cellular immunofluorescence. Compared with that in the control group, the fluorescence signal intensity of p66Shc protein at the 4- to 8-cell stage after microinjection of siRNA-a, siRNA-b, or siRNA-c was significantly reduced (p<0.05), and the fluorescence signal after microinjection of siRNA-b showed the lowest intensity (Figure 2B and C). Therefore, subsequent interference experiments used siRNA-b molecules to knock down p66shc.

### Knockdown of p66Shc improves the developmental potential of sheep embryos

**Figure 3:**
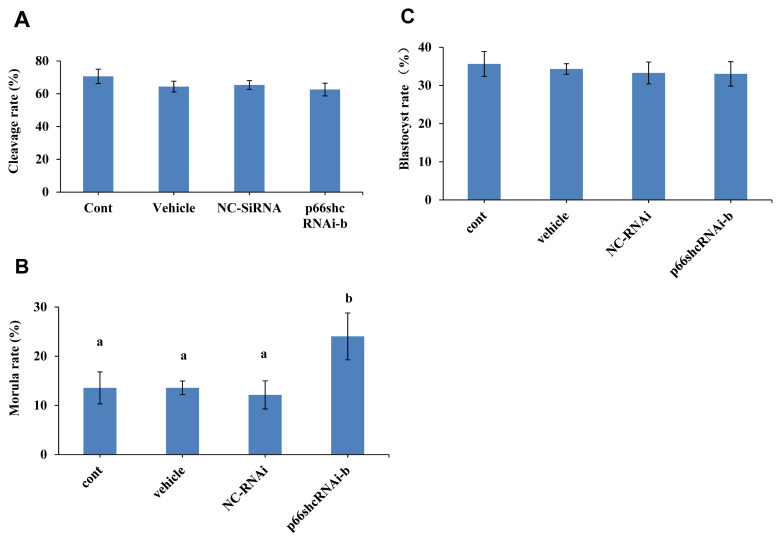
Effect of RNAi-mediated knockdown of p66Shc on embryo developmental potential. The cleavage rate (A), morula rate (B) and blastocyst rate (C) of sheep embryos in the noninjection group, the RNase-free water (vehicle) group, the negative control siRNA group and the siRNA-b group were compared. All data are shown as the mean±standard error of the mean. ^a,b^ Different lowercase letters indicate significant differences (p<0.05).

Whether p66Shc knockdown affects the developmental potential of sheep embryos was next determined. The results showed no significant difference in the cleavage rate and blastocyst rate of sheep embryos among the group microinjected with siRNA-b (63% and 36%), the uninjected group (70% and 34%), the group microinjected with RNase-free water (64% and 33%) and the group microinjected with NC-siRNA (65% and 33%) (p>0.05) (Figure 3A and C). However, the morula rate (24%) of the group microinjected with siRNA-b was significantly higher (p<0.05) than that of the control groups (noninjected group, 13%; group injected with RNase-free water, 13%; and group injected with NC-siRNA, 12%) (Figure 3B). These results suggest that p66shc knockdown significantly increases the potential of embryos to cross the arrest stage towards morula development.

### p66Shc knockdown reduces reactive oxygen species production

**Figure 4:**
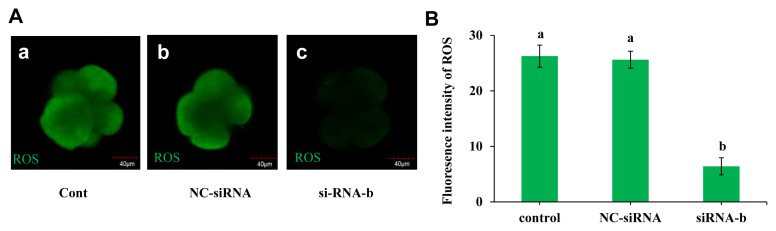
Effect of RNAi-mediated knockdown of p66Shc on the generation of embryonic reactive oxygen species (ROS). (A) Representative confocal images showing changes in ROS levels in the uninjected group (control group), the RNase-free water (vehicle)-injected group and the siRNA-b group. Scale bar = 40 μm. (B) Histogram showing the quantification of ROS intensity in embryos from different groups (n = 30 for each group). All data are shown as the mean±standard error of the mean. ^a,b^ Different lowercase letters indicate significant differences (p<0.05).

The effect of p66Shc knockdown on the production of ROS was examined, and the results are shown in Figure 4A and B. The ROS level in the group microinjected with siRNA-b was significantly lower than those in the noninjected group and the group microinjected with NC-siRNA (p<0.05). However, there was no significant difference between the noninjected group and the NC-siRNA group (p>0.05). This result indicated that p66shc knockdown significantly reduced embryonic ROS production, implying that p66Shc induces the OS signalling pathway by mediating ROS production.

### p66Shc knockdown reduces the production of the DNA damage marker 8-OHdG

**Figure 5:**
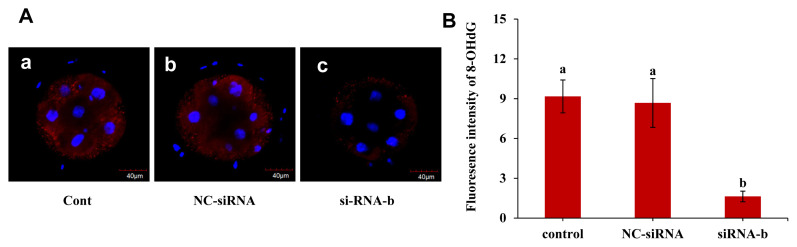
Effect of RNAi-mediated knockdown of p66Shc on the DNA damage marker 8-OHdG. (A) Representative confocal images showing changes in the levels of the DNA damage marker 8-OHdG in the uninjected group (control group), the RNase-free water (vehicle)-injected group and the siRNA-b group. Scale bar = 40 μm. (B) Histogram showing the quantification of 8-OHdG intensity in embryos from different groups (n = 30 for each group). All data are shown as the mean±standard error of the mean. ^a,b^ Different lowercase letters indicate significant differences (p<0.05).

The effect of p66Shc knockdown on the DNA oxidative damage marker 8-OHdG was examined, and the results are shown in Figure 5A and B. The fluorescence intensity of 8-OHdG in the group microinjected with siRNA-b was significantly lower than that in the uninjected group and the group microinjected with NC-siRNA at the 4- to 8-cell stage (p<0.05). However, the fluorescence intensity of 8-OHdG in the group microinjected with NC-siRNA was not significantly different from that in the noninjected group (p>0.05). This result indicated that p66shc knockdown significantly reduced the content of 8-OHdG, a biomarker of oxidative damage, suggesting that p66Shc is involved in oxidative damage mediated by ROS production in sheep embryos.

### p66Shc ablation increases the abundance of MnSOD in sheep embryos

**Figure 6:**
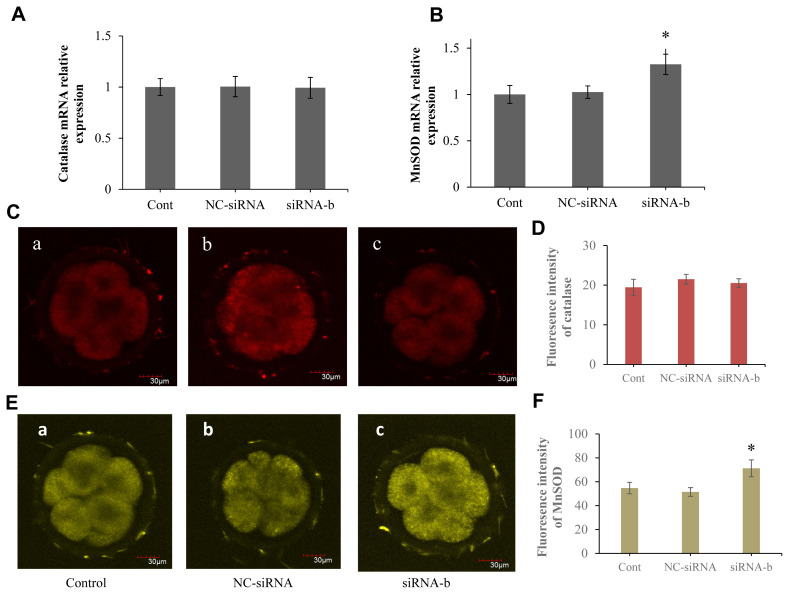
Effects of RNAi-mediated p66Shc knockdown on the expression of catalase and manganese superoxide dismutase (MnSOD). (A, B) Real-time polymerase chain reaction quantification of catalase and MnSOD mRNA levels in four- and eight-cell embryos from different groups (n = 30 for each group). (C, D) Representative confocal images showing changes in catalase and MnSOD protein in four- and eight-cell embryos from different groups (n = 30 for each group). Scale bar = 30 μm. (E, F) Histogram showing the quantification of catalase and MnSOD intensity in embryos from different groups (n = 30 for each group). All data are shown as the mean±standard error of the mean. * Means are significantly different (p<0.05).

The effect of p66Shc knockdown on antioxidant defence in sheep embryos was further explored. RT–qPCR and immunofluorescence staining were performed to examine the mRNA transcript levels and protein expression of catalase and MnSOD in sheep preimplantation embryos. However, no statistically significant differences were observed between the p66shc-knockdown group and the other catalase groups at the transcriptional and protein levels (Figure 6A, C, and D). Strikingly, we found that the expression of MnSOD in the p66shc-knockdown group was significantly higher than that in other experimental groups at both the transcriptional and protein levels (Figure 6B, E, and F), indicating the critical role of MnSOD in antioxidant defence during early sheep development. These results suggest that p66shc-knockdown embryos may exert antioxidant defence functions mainly through the MnSOD-ROS pathway.

## DISCUSSION

Studies of somatic cells have shown that p66Shc, as an oxidoreductase, plays an important role in the regulation of ROS-mediated OS, cell proliferation and apoptosis [9,10]. In a mouse model, p66Shc deletion mutants have been shown to enhance cellular resistance to ROS-induced OS [18] and resist age-related diseases such as endothelial dysfunction [19], cardiovascular diseases [20] and alcohol-induced liver disease [21]. Although the physiological role of p66Shc in somatic cells and knockout mice has been extensively studied, little is known about the redox function of p66Shc during sheep preimplantation embryonic development. In the present study, we revealed the temporal expression characteristics of p66Shc mRNA and protein and the translation and localization of a functional Ser36-phosphorylated p66Shc protein during early embryonic development. In addition, the results suggest that p66Shc is involved in the regulation of OS in early embryos and that *p66Shc* gene silencing can increase the resistance of embryos to OS and improve the developmental ability of embryos *in vitro*.

In recent years, with the expansion of the sheep *in vitro* production scale and the need for scientific research, a large proportion of oocytes have been enabled to successfully complete *in vitro* maturation, but developmental arrest or apoptosis occurs after fertilization [22,23]. The reason for this is largely related to the OS caused by redox imbalance during embryonic development *in vitro* [24,25]. It has been found that p66Shc is an important protein for intracellular redox homeostasis that participates in the regulation of cellular mitochondrial ROS metabolism [9,26]. To explore the expression characteristics of p66Shc in early preimplantation embryos, we investigated the functional role of p66Shc in a particular stage. In the present study, p66Shc mRNA expression showed an inverted bell-shaped pattern. Specifically, p66Shc mRNA was stored in oocytes and degraded during the maternal–embryonic transition. p66Shc mRNA was significantly upregulated at the morula and blastocyst stages after EGA. Previous studies have shown that RNA-mediated knockdown of p66Shc alters the spatiotemporal expression of cell lineage-associated transcription factors (e.g., OCT3/4, NANOG, and GATA4) in the inner cell mass (ICM) of the mouse blastocyst [27]. p66Shc transcript is upregulated at the blastocyst stage, indicating that p66Shc may also have an important physiological function other than promoting apoptosis and embryo arrest. It is possible that p66Shc expression is carefully regulated during preimplantation development, such that both abnormally high and low p66Shc expression levels are detrimental to the embryo. Therefore, further work must be performed to determine the mechanism of p66Shc function during preimplantation development and its implications for post-implantation development. In a mouse model study, it was found that p66Shc posttranslational modification plays an important role in proapoptotic activity [28]. For example, phosphorylation at Ser36 in the CH2 domain of the *p66Shc* gene is indispensable for peroxide- or UV-induced apoptosis [18,28]. Interestingly, immunofluorescence staining showed that Ser36-phosphorylated p66Shc was localized only in the cytosolic region of the blastomeres at the 2 to 8 stage but was localized mainly in the nucleus after EGA. These results suggest a specific role of p66Shc in preimplantation development. The results also showed a gradual translocation of phosphorylated (Ser36) p66Shc protein from the cytoplasm to the nuclear region, suggesting a stage-specific role of phosphorylated (Ser36) p66Shc in the EGA process, but further investigation is needed.

RNA interference is a common experimental method for the study of sequence-specific gene silencing in mammalian oocytes and early embryos [29,30]. In this study, all siRNA molecules were microinjected into the cytoplasm of zygotes. Designing multiple target sequences for the same RNA molecule is an effective means to verify gene interference efficiency. p66Shc is one of the largest members of the SHC adaptor protein family and exists in two isoforms, p46Shc and p52Shc. p66Shc differs from other isoforms in that p66Shc has an additional CH domain at the N-terminus, called the CH2 domain [31,32]. In this experiment, interfering molecules (siRNA-a, siRNA-b, siRNA-c) targeting different regions of the specific CH2 domain of p66Shc were designed and synthesized, and a negative control interfering molecule (NC-siRNA) was synthesized as a control. In the interference experiments, the results showed that the three siRNA molecules (siRNA-a, siRNA-b, and siRNA-c) could induce significant downregulation of p66Shc mRNA and protein compared with the expression in the uninjected group and the RNase-free water-injected and negative control siRNA-injected groups. However, siRNA-b was the most efficient and effective siRNA. Therefore, siRNA-b was selected as the interfering molecule to be used in the subsequent interference experiments.

Previous studies have shown that permanent embryo arrest frequently occurs during EGA in cattle and sheep primarily at the 8- to 16-cell stage. Embryos are vulnerable to OS at this stage [7,33]. The effect of microinjection of the p66Shc siRNA molecule on embryonic development was investigated, the results showed that knockdown of p66Shc had no effect on the cleavage rate and blastocyst rate but that it promoted development to the morula stage by overcoming embryo development arrest. These results indicate that RNAi-mediated silencing of p66Shc could enhance morula formation in sheep.

It is generally believed that p66Shc plays a key role in the process of mitochondrial ROS production. Under normal physiological conditions, intracellular ROS levels are in metabolic equilibrium. Once ROS metabolic imbalance occurs, OS is initiated, and ROS are potent inducers of oxidative damage [34,35]. We next investigated whether p66Shc is involved in OS and oxidative damage mediated by ROS production in sheep embryos by investigating the effect of p66Shc knockdown on ROS generation and DNA oxidative damage during sheep preimplantation development. The results of this study showed that p66Shc knockdown significantly reduced the generation of ROS. 8-Hydroxy-2 deoxyguanosine is often used as a biomarker of DNA oxidative damage due to hydroxyl radical attack at C8 of guanine [36]. The results showed that the levels of the DNA oxidative damage marker 8-OHDG were also significantly decreased by knockdown of p66Shc. Cellular ROS toxicity can be eliminated by antioxidant enzymes such as MnSOD and catalase [37]. In this study, RT–qPCR and immunofluorescence were used to detect the expression of MnSOD and catalase, and the results showed that RNAi-mediated p66Shc knockdown significantly increased the expression of MnSOD, suggesting that MnSOD-mediated ROS scavenging plays an important role in cell homeostasis. These results indicate that p66Shc is involved in the regulation of OS in early embryos and that *p66Shc* gene silencing can increase the resistance of embryos to OS and improve the developmental ability of embryos *in vitro*.

## CONCLUSION

In conclusion, the results of the present study provide a comprehensive spatiotemporal expression pattern of p66Shc and Ser36-phosphorylated p66Shc during sheep early embryonic development. We reveal that knockdown of p66Shc reduces endogenous p66Shc mRNA and protein levels. Moreover, siRNA-mediated depletion of p66Shc causes increased developmental potential to the morula stage, probably by regulating the MnSOD-ROS signalling pathway during EGA.

## Figures and Tables

**Table 1:** Molecule sequence information of p66Shc siRNA

Molecule	siRNA Sense	siRNA Antisense
siRNA-a	GCCCAAGUACAACCCACUUTT	AAGUGGGUUGUACUUGGGCTT
siRNA-b	GCAGUCAUGCUGGACUCAGTT	CUGAGUCCAGCAUGACUGCTT
siRNA-c	ACCACCCUGUGCUCCUUCUTT	AGAAGGAGCACAGGGUGGUTT
Negative control	UUCUCCGAACGUGUCACGUTT	ACGUGACACGUUCGGAGAATT

**Table 2:** Oligonucleotide primer sequences used in quantitative real-time polymerase chain reaction

Gene	Gene ID	Annealing temp (°C)	Sequence (5′–3′)	Product length (bp)
*p66Shc*	101113548	60	F: CGGGGTTTCCTACTTGGTT	169
			R: ACGGCTACAGGGCTTTCTC	
*Catalase*	100307035	60	F: GCCTGTGTGAGAACATTGCG	121
			R: TCCAAAAGAGCCTGGATGCG	
*MnSOD*	780457	60	F: ATTGCTGGAAGCCCATCAAAC	118
			R: AGCAGGGGGATAAGACCTGT	
*β-Actin*	443052	60	F: GTCATCACCATCGGCAATGA	88
			R: CGTGAATGCCGCAGGATT	

*MnSOD*, manganese superoxide dismutase.
